# Purified vitexin compound 1, a new neolignan isolated compound, promotes PUMA‐dependent apoptosis in colorectal cancer

**DOI:** 10.1002/cam4.1769

**Published:** 2018-11-06

**Authors:** Jingfei Chen, Juchang Zhong, Yeying Liu, Yuan Huang, Fei Luo, Yingjun Zhou, Xi Pan, Shousong Cao, Lingling Zhang, Yingjie Zhang, Jiangang Wang

**Affiliations:** ^1^ Department of Internal Medicine The Third Xiangya Hospital Central South University Changsha China; ^2^ College of Biology Hunan University Changsha China; ^3^ Department of Laboratory Medicine Xiangya School of Medicine Central South University Changsha China; ^4^ Department of Obstetrics and Gynecology Xiangya Hospital Central South University Changsha China; ^5^ Department of Cardiology The Second Xiangya Hospital Changsha China; ^6^ School of Pharmaceutical Science Central South University Changsha China; ^7^ Department of Oncology The Third Xiangya Hospital Central South University Changsha China; ^8^ Department of Pharmacology School of Pharmacy Southwest Medical University Luzhou China; ^9^ Shenzhen Institute Hunan University Shenzhen China

**Keywords:** apoptosis, Bcl‐2‐associated X protein, colorectal cancer, p53, PUMA, vitexin compound 1

## Abstract

Purified vitexin compound 1 (VB1, a neolignan isolated and extracted from the seed of Chinese herb Vitex negundo) is an effective antitumor agent and exhibits promising clinical activity against various cancers including colorectal cancer. However, it remains unknown about the precise underlying mechanism associated with the antitumor effect of VB1 and how it triggers apoptosis in cancer cells. Here, we demonstrated that VB1 promoted apoptosis via p53‐dependent induction of p53 upregulated modulator of apoptosis (PUMA) and further to induce Bax (Bcl‐2‐associated X protein) activation and mitochondrial dysfunction in colon cancer HCT‐116 and LoVo cells. Deficiency in p53, PUMA, or Bax abrogated VB1‐induced apoptosis and promoted cell survival in HCT‐116 cells. Furthermore, the combination of VB1 with chemotherapeutic drugs 5‐fluorouracil (5‐FU) or NVP‐BZE235 resulted in a synergistic antitumor effect via PUMA induction in HCT‐116 cells. VB1 significantly suppressed the cell proliferation of wild‐type (WT) HCT‐116 and LoVo cells in vitro and tumor growth in vivo. The results indicate that p53/PUMA/Bax axis plays a critical role in VB1‐induced apoptosis and VB1 may have valuable clinical applications in cancer therapy as a novel anticancer agent used alone or in combination with other chemotherapeutic drugs.

## 1 INTRODUCTION

Colorectal cancer is the third most frequent malignancy and the third cause of cancer‐related deaths.^1^ It affected approximately 95 270 people and resulted in 49 190 deaths in the United States in 2016.^2^ Although the treatment of colorectal cancer has made substantial improvement in the past decades, patients with metastatic colon cancer have only about 3 years of median overall survival,^3^, ^4^ because most patients eventually develop acquired drug resistance.^5^, ^6^, ^7^, ^8^ Drug resistance and dose‐limiting toxicity limit the efficacy of clinical response to chemotherapy.^9^, ^10^ Therefore, there is an urgent need to develop novel chemical entities that have better anticancer activity, less toxicity, or complement current chemotherapeutic drugs to improve their anticancer efficacy against colorectal cancer.

The Bcl‐2 family members are the central signaling moleculars of mitochondrial‐mediated apoptosis. The BH3‐only subfamily proteins, composed of at least 10 members, are critical regulators involved in response to different and overlapping signals. As a BH3‐only Bcl‐2 family member, p53 upregulated modulator of apoptosis (PUMA) serves as a vital apoptosis initiator in cancer cells.^11^ It can be transcriptionally activated by p53 in response to DNA‐damaging agents.^12^, ^13^, ^14^ The activation of PUMA by nongenotoxic stimuli is p53‐independent and mediated by different transcription factors, including p53 homologue p73,^15^, ^16^ forkhead box O3a (FoxO3a),^17^, ^18^, ^19^, ^20^ and nuclear factor‐kappa B (NF‐κB).^21^, ^22^ Upon induction, PUMA facilitates mitochondrial outer membrane permeabilization and caspase activation cascade to regulate Bcl‐2‐associated X protein (Bax) activity.^23^, ^24^, ^25^, ^26^, ^27^, ^28^, ^29^, ^30^ PUMA‐deficient colorectal cancer cells eliminate mitochondrial apoptosis caused by various stimuli.^31^, ^32^, ^33^, ^34^, ^35^, ^36^ However, overexpression of PUMA accelerates apoptosis in various cancer cells.^37^, ^38^

**Figure 1:**
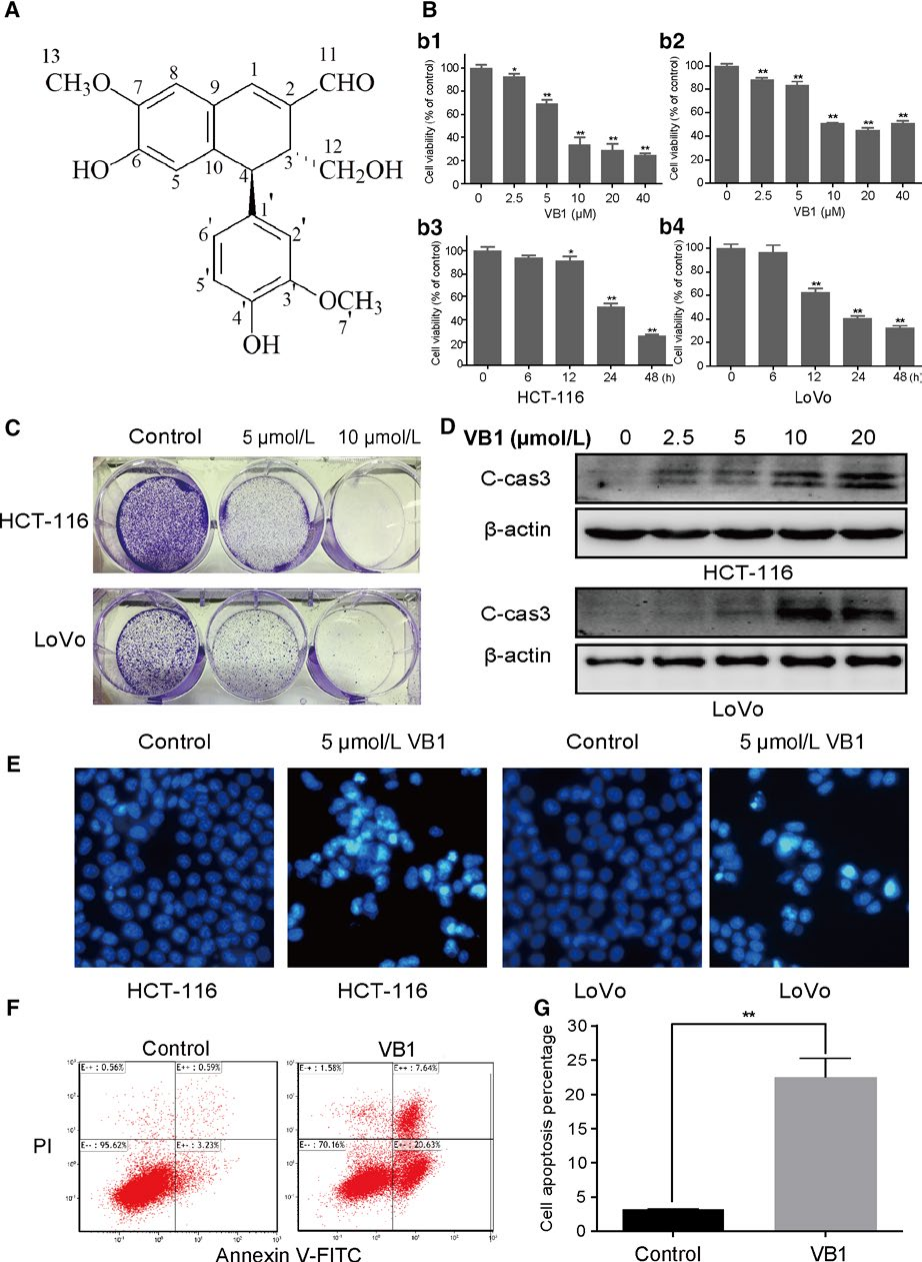
VB1 inhibits the growth and induces apoptosis of human colon cancer cell HCT‐116 and LoVo cells. A, Chemical structure of VB1 (MW. 354). B, Cell viability was analyzed by Cell Counting Kit‐8 at indicated time after VB1 treatment. C, Colony formation was analyzed by crystal violet staining of attached cells on day 14 after HCT‐116 (upper panel) and LoVo (lower panel) cells were treated with VB1 for 24 h. D, The expression of cleaved caspase 3 was analyzed by Western blotting. E, The morphological examination of apoptosis was analyzed by Hoechst 33342 staining after cells were treated with VB1 for 24 h. F and G, Cell apoptosis was analyzed by fluorescence‐activated cell sorting (FACS) analysis after HCT‐116 cells were treated with 10 μmol/L VB1 for 24 h. The percentage of apoptotic cells were calculated from FACS analysis. Data represent the mean ± SD of three independent experiments. *P <* *0.05, **P <* *0.01 vs untreated control cells

Lignans extensively exist in fruits, cereals, and vegetables, etc,.^39^ Numerous evidence has demonstrated that plant lignans can suppress cancer cell proliferation and may reduce the risk of cancers.^39^, ^40^ Vitex negundo, a Chinese herb, has been widely used for treatment of cough, asthma, and arthritis as a folk medicine in China because of its anti‐inflammatory effect. We have isolated a mixture of neolignan compounds from the seeds of Vitex negundo named EVn‐50 and further purified fifteen lignan compounds from EVn‐50, which showed the effects of apoptosis induction and tumor growth suppression.^41^ Among them, vitexin compound 1 (VB1,6‐hydroxy‐4‐(4‐hydroxy‐3‐methoxyphenyl)‐3‐hydro‐methyl‐7‐methoxy‐3,4‐dihydro‐2‐naphthaldehyde, Figure 1A) is the main lignan, accounting for 38% constitutions of EVn‐50.^41^ Previous studies have shown that the VB1 induced apoptosis in various cancers including colorectal cancer and its hypotoxicity and high efficiency make it a more ideal antitumor drug.^42^, ^43^, ^44^ However, it remains to be clarified for the underlying mechanisms of apoptosis induction by VB1 in colorectal cancer cells.

In this study, we assessed the effects of VB1 on cell proliferation inhibition and apoptosis induction in human colorectal cancer in vitro and in vivo. We also studied the expression, function, and molecular mechanism of PUMA during VB1‐induced apoptosis in both wild‐type (WT) and p53, PUMA, and Bax knockout (KO) cells. Furthermore, we investigated the combinational therapy of VB1 plus 5‐fluorouracil (5‐FU) or VB1 plus NVP‐BZE235 in colon cancer cells, and demonstrated the indispensability of PUMA/Bax axis in these processes. Finally, the antitumor activity of VB1 was analyzed in the xenograft model in vivo, and showed a positive therapeutic effect. For the first time, our results revealed that VB1 suppressed colorectal cancer growth via p53/PUMA/Bax signaling pathway, which makes VB1 as a potential antitumor drug in clinical application in the future.

## 2 MATERIALS AND METHODS

### 2.1 Cell Culture and drug treatments

All the cell lines (HCT‐116 WT, HCT‐116 p53‐KO, HCT‐116 PUMA‐KO, HCT‐116 BAX‐KO and LoVo) were obtained from American type culture collection (ATCC) and were cultured in McCoy's 5A modified media (Invitrogen), supplemented with 10% FBS, 100 units/mL penicillin, and 100 μg/mL streptomycin. Cells were maintained in a 37°C at 5% CO_2_ incubator. VB‐1 was purified from EVn‐50, a mixture of lignan compounds from Vitex negundo seed, as described previously.^41^ For treatment, various concentrations of VB1 (2.5, 5, 10, 20, and 40 μmol/L), 5‐FU (APP Pharmaceuticals, 100, 200, and 400 μmol/L), and NVP‐BZE235 (LC Laboratories, 200, 400, and 800 nmol/L) or their combination (5 μmol/L VB1 + 100 μmol/L 5‐FU or 5 μmol/L VB1 + 200 nmol/L NVP‐BZE235) were added in the medium directly before detection, while the same volume of medium was used as control.

### 2.2 Transfection studies

The plasmids of expressing PUMA and GFP‐Bax were kindly provided by Dr. Jian Yu.^14^ HCT‐116 cells were cultured in a 6‐well plate at a density of 10^6^/well. Cells were transfected with pGFP‐Bax or pPUMA following Polyjet^™^ transfection manufacturer's instructions. The medium was replaced with fresh culture medium after 8 hours. One day post‐transfection, cells were treated with medium (control) or 10 μmol/L VB1 for 24 hours followed by related experiments.

### 2.3 Western blotting

After drug treatment for indicated time, cell protein samples were extracted with radioimmunoprecipitation assay (RIPA) buffer [10 mmol/L Tris‐Cl (pH 8.0), 1 mmol/L EDTA, 0.5 mmol/L EGTA, 1% Triton X‐100, 0.1% sodium deoxycholate, 0.1% SDS, and 140 mmol/L NaCl]. Equivalent protein samples (30 μg protein extract was loaded on each lane) were separated by 10% SDS‐PAGE, transferred onto PVDF membranes (Millipore) and blocked with 5% nonfat milk for 1 hour at room temperature. The membranes were incubated with anti‐p53, p73, phospho‐Akt (S473), total‐Akt, PUMA, phospho‐FoxO3a, total‐FoxO3a, phospho‐p65 (S276 and S536), total‐p65 and cleaved caspase3 (Cell Signaling Technology), and β‐actin antibody (Santa Cruz) primary antibodies at 4°C overnight. Primary antibody was detected by binding horseradish peroxidase (HRP)‐conjugated anti‐rabbit or anti‐mouse secondary antibody with an ECL plus kit (Advansta, MenloPark, CA, USA). β‐actin was used as a loading control.

### 2.4 Xenograft mouse model

Five‐week‐old SPF Grade female *nu/nu* mice (Vital River Lab Animal Technology Co. Ltd., Beijing, China, Certificate No. SYXK2013‐0001) were housed in sterile microisolator cages (five per cage) with free access to water and food ad libitum. All animal experiments were carried out followed the protocols approved by Central South University Animal Use and Care Committee (Changsha, Hunan, China). 1 × 10^6^ cells were injected s.c. into both flanks of mice. Mice were administered by i.p. injection of VB1 40 mg/kg every other day for 2 weeks when tumors were measurable, whereas the same volumes of normal saline (NS) were used as vehicle control. Mice were euthanized when tumors reached ~1.0 cm^3^ (1000 mg) in size. Tissues of tumors were collected and examined. The protein was extracted using a Total Protein Extraction kit (Chemicon International, Temecula, CA, USA) and analyzed by Western Blotting.

### 2.5 Analysis of cell viability and apoptosis

Cells were cultured in 96‐well microplate at a density of 5 × 10^3^ cells/well for 24 hours. Cell viability was assessed with Cell Counting Kit‐8 (CCK‐8) (7Sea Biotech, Shanghai, China) at indicated time post‐treatment following the manufacturer's instructions. The absorbance value at 450 nm (OD450) was read with a 96‐well plate reader (DG5032, Hua Dong, Nanjing, China), to determine the cell viability.

For colony formation assay, cells were cultured in 6‐well plate at a density of 5 × 10^4^ cells/well for 24 hours. The cells were then treated with indicated concentrations of drugs and medium (control) for 24 hours. Medium was changed every 2 days. Colonies were visualized with crystal violet staining at Day 14.

For analysis of apoptosis by nuclear staining, cells were cultured in a 3.5‐cm dish, rinsed with phosphate‐buffered saline (PBS) twice and then 500 μL DMEM containing 5 μg Hoechst 33342 was added into the plates and incubated for 15 minutes in an incubator. Apoptosis was assessed through microscopic visualization of condensed chromatin and micronucleation. Apoptosis indices were calculated as the percentage of apoptotic cells among one hundred cells in a randomly selected portion. The positive rate of apoptotic cells was calculated by GD‐10.0 image analysis system.

### 2.6 Flow cytometry

HCT‐116 and LoVo cells were suspended in 1 × 10^6^ cells/mL, and 5 μL of Annexin V and propidium iodide staining solution were added to 300 μL of the cell suspension. After the cells were incubated at room temperature for 15 min in the dark, stained cells were assayed and quantified using a FACSort Flow Cytometer (Beckman Coulter, Brea, CA, USA). Cell debris was excluded from the analysis by an appropriate forward light scatter threshold setting. Compensation was used wherever necessary.

### 2.7 Co‐immunoprecipitation

HCT‐116 cells were cultured in 10‐cm dish at a density of 8 × 10^6^ cells/dish for 24 hours. Cells were then treated with 10 μmol/L VB1 for 24 hours, and the same volume of medium was used as control. Cells were harvested and lysed with lysis buffer (25 mmol/L HEPES, 125 mmol/L K‐acetate, 2.5 mmol/L‐acetate, 2 mmol/L DTT, 0.4% Tx‐100, 2X Phosphatase Inhibitor, Protease Inhibitor, Na Orthovanadate 400 μmol/L, pH = 7.2). To detect the interaction between PUMA and Bax, anti‐PUMA antibodies (~4 μL) were firstly added to 400 μL cell lysates and mixed on a rocker at 4°C for 4 hours. The immunocomplexes were captured by the addition of protein G/A‐agarose (Roche Applied Sciences) mixed at 1:10 ratio, followed by an additional 1 hour incubation. The beads were washed three times by PBS and then collected by centrifugation at 300 g for 5 minutes. After the final wash, the beads were mixed with 60 μL of 2× Laemmli sample buffer, heated at 100°C for 5 minutes, and analyzed by Western blotting.

### 2.8 GFP‐Bax translocation assay

HCT‐116 cells were cultured in a 3.5‐cm dish at a density of 10^6^/dish for 24 hours and then transfected with plasmid GFP‐Bax (conc. 902.4 ng/μL) following Polyjet^™^ transfection protocol (please see Transfection method section). One day post‐transfection (please see the 2.2 Transfection Studies), cells were treated with medium (control) and 10 μmol/L VB1 for 24 hours. After mitochondria were stained with MitoTracker ^®^Red CMXRos (Shanghai Yesen), the distribution pattern of GFP‐Bax with or without VB1 treatment was imaged under a Olympus FV1000 confocal microscope. Bax redistribution was examined by matching fluorescence emitted by GFP‐Bax (green) and MitoTracker (red). The cells, which exhibited intense punctate staining of GFP overlapping with the distribution of MitoTraker, were considered for mitochondrially localized Bax.

### 2.9 Statistical analysis

Statistical analysis was carried out using GraphPad Prism V software (GraphPad Software Inc., La Jolla, CA, USA). *P* value was calculated via either Student's *t* test or one‐way analysis of variance (ANOVA). Statistical significance was defined as *P*‐value <0.05 (marked as *).

## 3 RESULTS

### 3.1 VB‐1 suppressed proliferation and induced apoptosis in colon cancer cells

VB1 (Figure 1A) significantly inhibited the proliferation of HCT‐116 and LoVo cells in a concentration‐ and time‐dependent manner (Figure 1B), and the results were further confirmed by colony formation assay (Figure 1C). Next, the effect of VB1 on cell apoptosis was evaluated by Western blotting. The cleaved caspase 3, a critical apoptotic marker, was markedly increased after the cells were treated with 2.5, 5, 10, or 20 μM of VB1, respectively (Figure 1D). Chromatin condensation was obviously shown in HCT‐116 and LoVo cells at 24 hours after VB1 treatment (Figure 1E). FACS analysis also showed that cell apoptosis increased remarkably in VB1‐treated group compared with control group in HCT‐116 cells (*P *<* *0.05) (Figure 1F,G).

### 3.2 VB1 induced p53‐dependent PUMA expression

**Figure 2:**
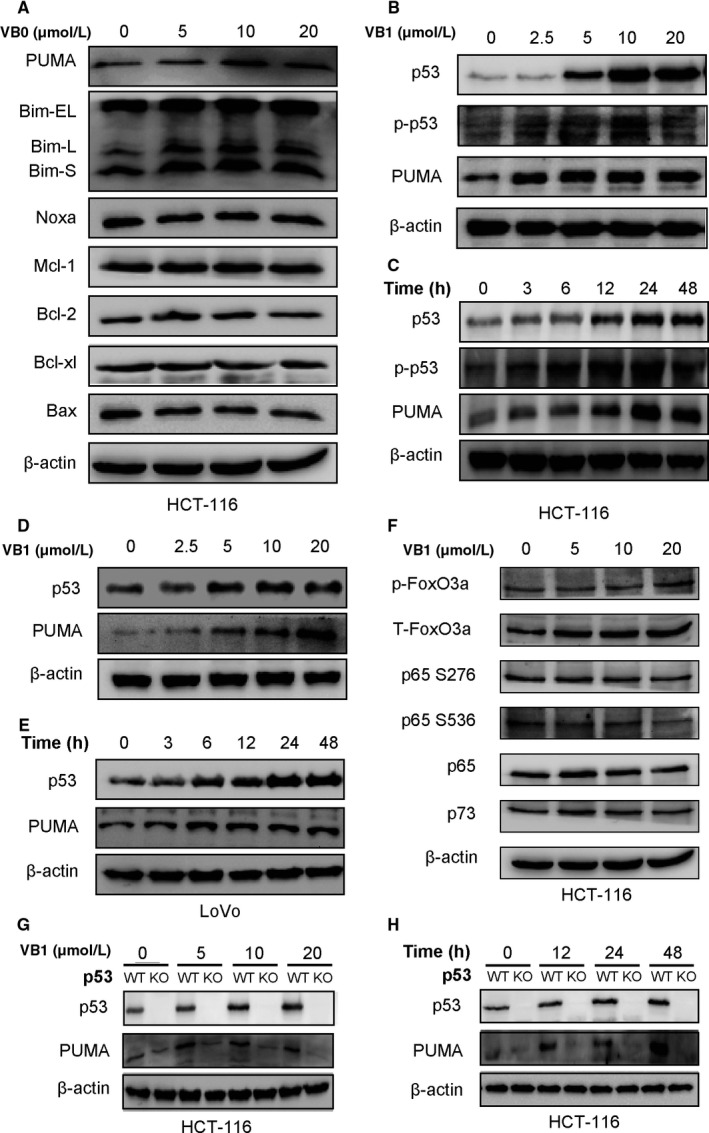
Effects of VB1 on apoptotic related proteins expressions in HCT‐116, p53‐KO HCT‐116 or LoVo cells by Western blotting. A, The expressions of PUMA, Bim‐EL, Bim‐L, Bim‐S, Noxa, Mcl‐1, Bcl‐2, Bcl‐xl, and Bax were analyzed after HCT‐116 cells were treated with VB1 for 24 h. B, The expressions of p53, p‐p53, and PUMA were analyzed after HCT‐116 cells were treated with VB1 for 24 h. C, The expressions of p53, p‐p53, and PUMA were analyzed after HCT‐116 cells were treated with 10 μmol/L VB1 for indicated time. D, The expressions of p53 and PUMA were analyzed after LoVo cells were treated with VB1 for 24 h. E, The expressions of p53 and PUMA were analyzed after LoVo cells were treated with 10 μmol/L VB1 for indicated time. F, The expressions of p‐FoxO3a, T‐FoxO3a, p65 S276, p65 S536, p65, and p73 were analyzed after HCT‐116 cells were treated with VB1 for 24 h. G, The expressions of p53 and PUMA were analyzed after WT and p53‐KO HCT‐116 cells were treated with VB1 for 24 h. H, The expressions of p53 and PUMA were analyzed after WT and p53‐KO HCT‐116 cells were treated with 10 μmol/L VB1 for indicated time

We first examined the effect of VB1 on the major members of Bcl‐2 family at the protein level by Western Blotting. VB1 concentration‐dependently increased the expression of PUMA and slightly increased the expression of Bim. However, VB1 did not affect the expressions of other Bcl‐2 family members such as Bcl‐xl, Mcl‐1, Bcl‐2, and Bax (Figure 2A). PUMA induction was dose‐ and time‐dependent, and VB1 could induce it at the concentration as low as 2.5 μmol/L and as early as 3 hours (Figure 2B,C). The results were also confirmed in LoVo cells (Figure 2D,E). We then further examined the transcription factors that transactivate PUMA, such as FoxO3a, p73, p65, and p53 to investigate their role in VB1‐induced PUMA expression. The results showed VB1 did not change the expression of p‐FoxO3a, FoxO3a, p65 S276, p65 S536, p65 and p73 (Figure 2F), and no translocation of p65 or FoxO3a occurred by immunofluorescence analysis after VB1 treatment (Figure S2). The data indicate that they play little or no role in PUMA induction. Both HCT‐116 and LoVo cells contain WT p53, we found that VB1 treatment resulted in p53 upregulation in these cells (Figure 2B‐E). Furthermore, the effect of VB1 on PUMA induction was almost completely blocked in p53‐KO cells, compared to that of the parental cells (Figure 2G,H). These results imply that PUMA induction by VB1 is mediated by p53‐dependent mechanism.

### 3.3 P53/PUMA pathway played a vital role in VB1‐induced cell apoptosis

**Figure 3:**
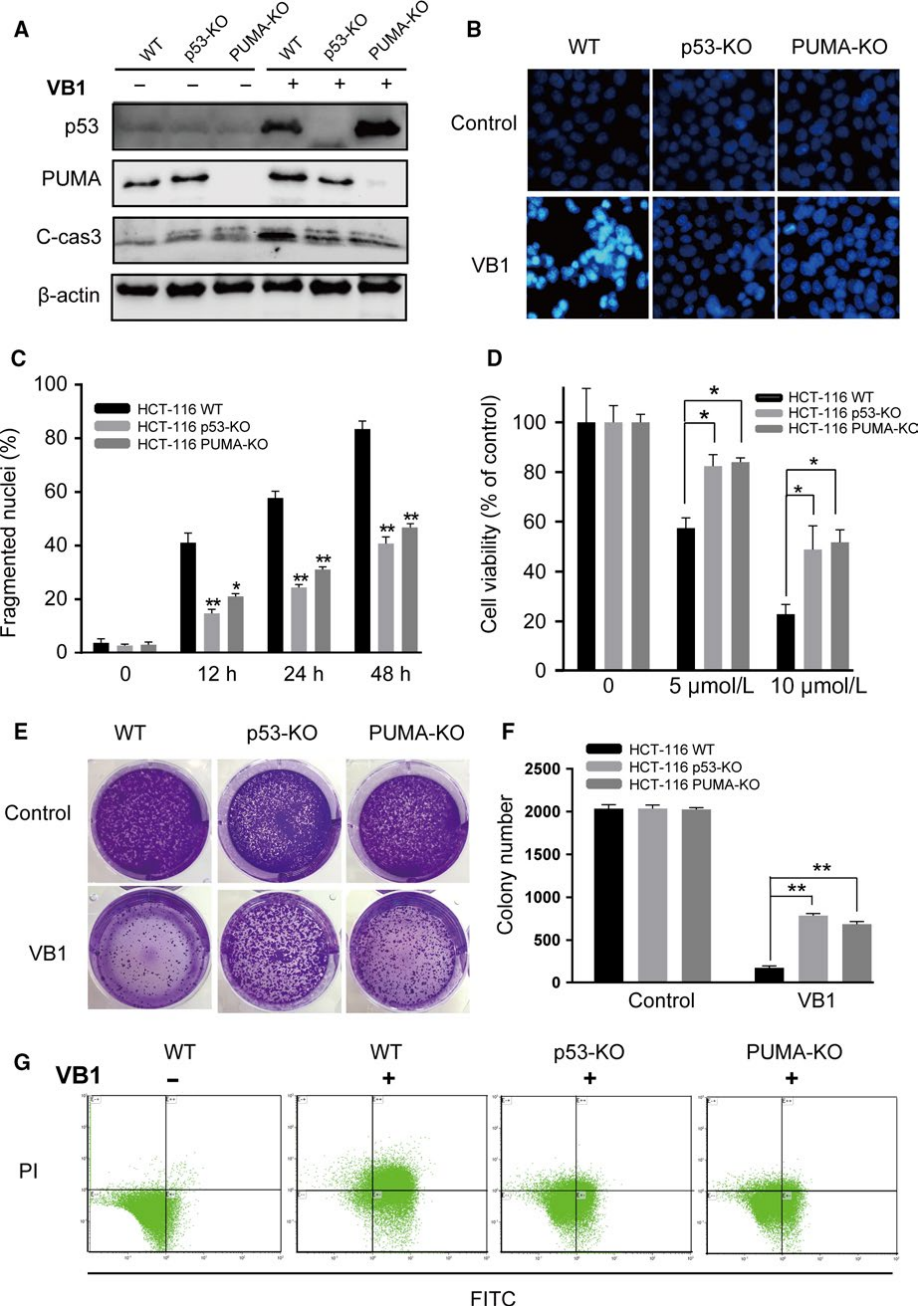
P53/PUMA axis is indispensible in VB1‐induced apoptosis in HCT‐116 cells. A, The expressions of p53, PUMA and cleaved caspase3 were analyzed by Western blotting after the cells were treated with 10 μmol/L VB1 for 24 h. B, The morphological examination of apoptosis was analyzed by Hoechst 33342 staining assay after the cells were treated with 10 μmol/L VB1 for 24 h. C, Condensed and fragmented nuclei were counted for apoptosis from morphological examination. D, Cell viability was analyzed by CCK‐8 after the cells were treated with 5 or 10 μmol/L VB1 for 72 h. E and F, Colony formation assay was performed by seeding an equal numbers of WT, p53‐KO and PUMA‐KO HCT‐116 cells in 6‐well plates and treated with 10 μmol/L VB1 for 24 h, then the attached cells were stained with crystal violet on day 14. Representative pictures of colonies and quantification of colony numbers are shown from four independent experiments expressed as mean ± SD. *P <* *0.05 or **P <* *0.01, WT vs p53‐KO or PUMA‐KO cells. G, Effect of VB1 on the apoptotic induction in HCT‐116 cells by FACS analysis. WT, p53‐KO HCT‐116, and PUMA‐KO HCT‐116 cells were treated with 10 μmol/L VB1 for 24 h

To examine the activation of p53/PUMA pathway in VB1‐induced apoptosis, parallel studies have been performed in PUMA‐KO and p53‐KO HCT‐116 cells. The results showed p53 expression but not cleaved caspase 3 was upregulated by VB1 in PUMA‐KO HCT‐116 cells (Figure 3A), indicating that PUMA contributed to VB1‐induced apoptosis but did not affect expression of p53, and VB1 increased expressions of PUMA and cleaved caspase 3 in the WT but not in the p53‐KO HCT‐116 cells (Figures 3A and S3a), implying that p53 is indispensable for VB1‐induced PUMA upregulation and apoptosis. The chromatin condensation of p53‐KO and PUMA‐KO HCT‐116 cells was significantly lower than that of WT HCT‐116 cells detected by Hoechst 33342 staining after VB1 treatment. (Figure 3B,C). Furthermore, apoptotic resistance to VB1 was also confirmed by Annexin V/propidium iodide staining in p53‐KO and PUMA‐KO cells (Figure S3b,c). The cell viability and colony formation were also significantly increased in p53‐KO and PUMA‐KO HCT‐116 cells after VB1 treatment (Figure 3D‐F). Therefore, these results suggest that p53/PUMA pathway is necessary for VB1‐induced apoptosis in colon cancer HCT‐116 cells.

### 3.4 VB1 induced apoptosis via PUMA/Bax signaling pathway

**Figure 4:**
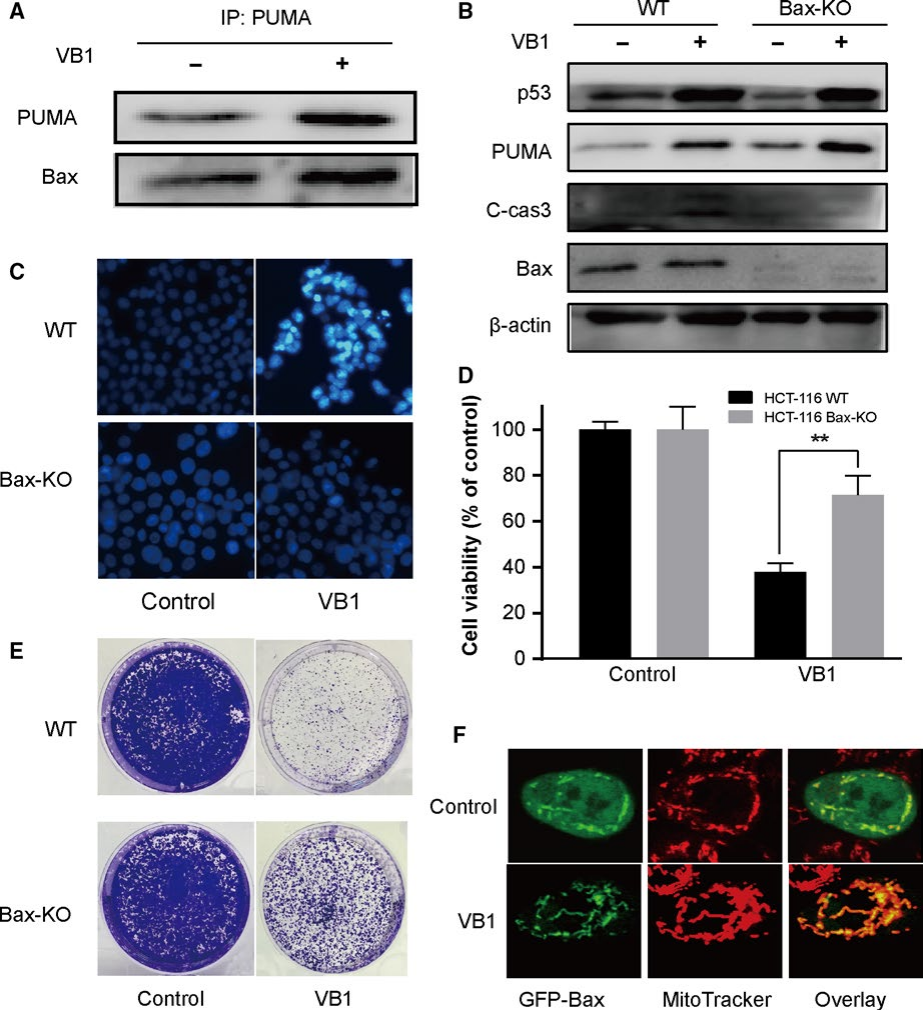
PUMA/Bax pathway is essential to VB1‐induced apoptosis in HCT‐116 cells. A, The interaction of PUMA and Bax was detected by Co‐immunoprecipitation (Co‐IP) with an anti‐PUMA antibody to pull down PUMA, and the amount of Bax binding to PUMA was analyzed by Western blotting. B, P53, PUMA, Bax, and cleaved caspase 3 were analyzed by Western blotting in WT and Bax‐KO HCT‐116 cells after 10 μmol/L VB1 treatment for 24 h. C, Morphological examination of apoptosis in WT and Bax‐KO HCT‐116 cells after 10 μmol/L VB1 treatment for 24 h by Hoechst 33342 staining assay. D, Cell viability was analyzed by CCK‐8 in WT and Bax‐KO HCT‐116 cells after 10 μmol/L VB1 treatment for 72 h. E, Colony formation assay was performed by seeding an equal number of WT and Bax‐KO HCT‐116 cells in 6‐well plates and treated with 10 μmol/L VB1 for 24 h, then the attached cells were stained with crystal violet on day 14. F, Bax translocation induced by VB1 in WT HCT‐116 cells. HCT‐116 cells were transfected with GFP‐Bax and stained with mitotracker with or without 10 μmol/L VB1 treatment. The data represent the mean ± SD of three to four independent experiments

We and others reported before that PUMA‐induced cell apoptosis was associated with activation of Bax directly and indirectly.^29^, ^30^ Here, we further investigated the relationship between PUMA and Bax in VB1 treated HCT‐116 cells. As shown in Figure 4A, there was a direct interaction between PUMA and Bax and both of them were significantly elevated by VB1. To clarify the role of Bax activation in VB1‐induced apoptosis, the expressions of p53 and PUMA as well as cleaved caspase 3 were investigated in the WT and Bax‐KO HCT‐116 cells following VB1 treatment. As shown in Figure 4B, the expressions of p53 and PUMA increased in both WT and Bax‐KO cells by VB1 (Figure 4B). However, cleaved caspase 3 was increased in WT HCT‐116 cells only whereas remained unchanged in Bax‐KO HCT‐116 cells after VB1 treatment (Figure 4B). Furthermore, chromatin condensation was significantly inhibited in Bax‐KO HCT‐116 cells (Figure 4C). Consistent results were also obtained by cell viability (Figure 4D) and colony formation assays (Figure 4E). Finally, we observed VB1 induced the mitochondrial translocation of GFP‐Bax in HCT‐116 cells through confocal microscopy (Figure 4F) to further confirm Bax activation by VB1. Therefore, the data indicate that Bax is an essential initiator for cell apoptosis induced by VB1.

### 3.5 VB1 synergized with 5‐FU and NVP‐BZE235 to induce PUMA‐dependent apoptosis

**Figure 5:**
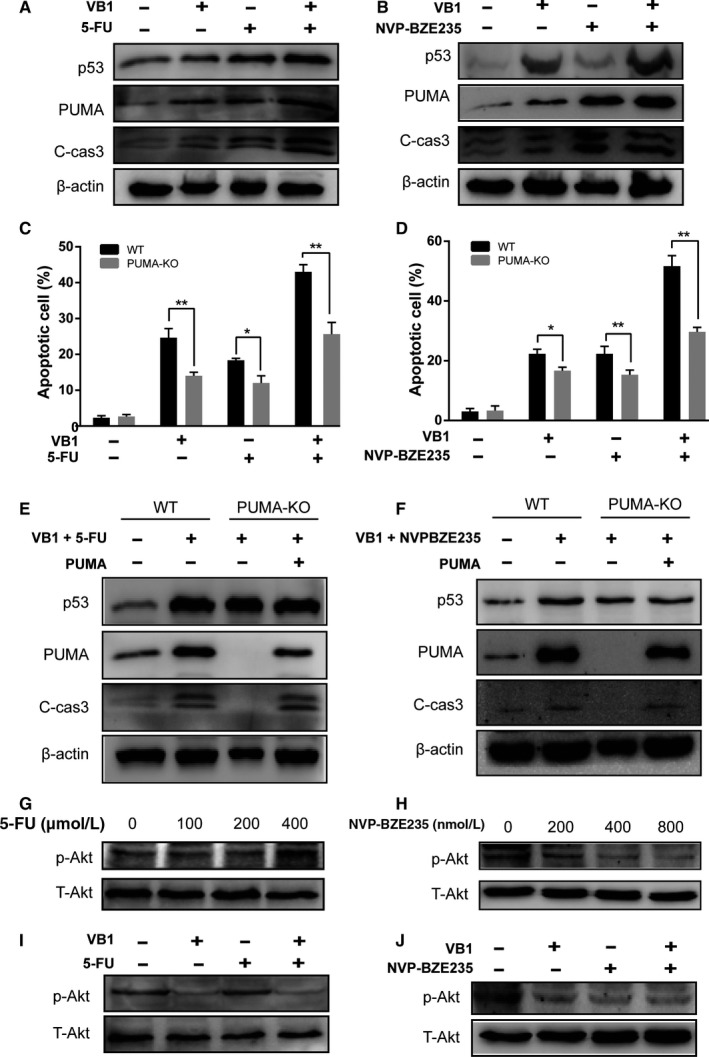
Combination of VB1 with 5‐FU or NVP‐BZE235 induced colon cancer cell apoptosis is PUMA‐dependent. A, The expressions of p53, PUMA, and C‐cas3 were analyzed by Western blotting after HCT‐116 cells were treated with 10 μmol/L VB1 and 200 μmol/L 5‐FU alone or in combination for 24 h. B, The expressions of p53, PUMA, and C‐cas3 were analyzed by Western blotting after HCT‐116 cells were treated with 10 μmol/L VB1 and 400 nmol/L NVP‐BZE235 alone or in combinations for 24 h. C, Apoptosis was analyzed by Hoechst 33342 staining assay after WT or PUMA‐KO HCT‐116 cells were treated with 10 μmol/L VB1 and 200 μmol/L 5‐FU alone or in combination for 24 h. *P <* *0.05 or **P <* *0.01, WT vs PUMA‐KO cells. D, WT or PUMA‐KO HCT‐116 cells were treated with 10 μmol/L VB1 and 400 nmol/L NVP‐BZE235 alone or in combination for 24 h, and apoptosis was analyzed by Hoechst 33342 staining assay. *P <* *0.05 or **P <* *0.01, WT vs PUMA‐KO cells. E, The expressions of p53, PUMA, and C‐cas3 were analyzed after HCT‐116 cells were treated with 10 μmol/L VB1 or 200 μmol/L 5‐FU alone or in combination for 24 h. F, The expressions of p53, PUMA and C‐cas3 were analyzed after HCT‐116 cells were treated with 10 μmol/L VB1 or 400 nmol/L NVP‐BZE235 alone or in combination for 24 h. G and H, The expressions of p‐Akt and T‐Akt were analyzed after HCT‐116 cells were treated with 0, 100, 200, or 400 μmol/L 5‐FU (G) and 0, 200, 400, or 800 nmol/L NVP‐BEZ235 (H) for 24 h. I, The expressions of p‐Akt and T‐Akt were analyzed after HCT‐116 cells were treated with 10 μmol/L VB1 or 200 μmol/L 5‐FU alone or in combination for 24 h. J, The expressions of p‐Akt and T‐Akt were analyzed after HCT‐116 cells were treated with 10 μmol/L VB1 or 400 nmol/L NVP‐BZE235 alone or in combination for 24 h. All data represent the mean ± SD of four independent experiments

After we demonstrated the pro‐apoptotic role of PUMA and tumor growth inhibition by VB1, we further studied the effect of VB1 in combination with other chemotherapeutic drugs on apoptosis. We selected a DNA‐damaging drug 5‐FU and a novel dual PI3K/mTOR inhibitor NVP‐BZE235 for induction of PUMA expression and apoptosis in HCT‐116 cells (Figure S4a‐d). The results showed that VB1 combined with 5‐FU or NVP‐BZE235 led to higher expression of PUMA and cleaved caspase 3, compared to mono treatment (Figures 5A,B and S4e). In addition, combination therapy induced higher levels of apoptosis in WT HCT‐116 cells, but not in PUMA‐KO cells (Figure 5C,D). Moreover, the apoptotic effect of combination was completely restored by addition of exogenous PUMA (Figure 5E,F), implying PUMA is essential for the effect of combination treatment‐induced apoptosis, which is consistent with our previous study.^45^ 5‐FU greatly increased phospho‐Akt but NVP‐BZE235 reduced Akt phosphorylation (Figure S4f). However, phospho‐Akt was greatly reduced with the treatment of VB1 combined with 5‐FU or NVP‐BZE235 (Figure 5G,H). These data demonstrate that PUMA mediates the effect of chemosensitization of VB1 and robust induction of PUMA can enhance apoptosis, thereby improve the therapeutic efficacy.

### 3.6 VB1 inhibited colon cancer growth in vivo

**Figure 6:**
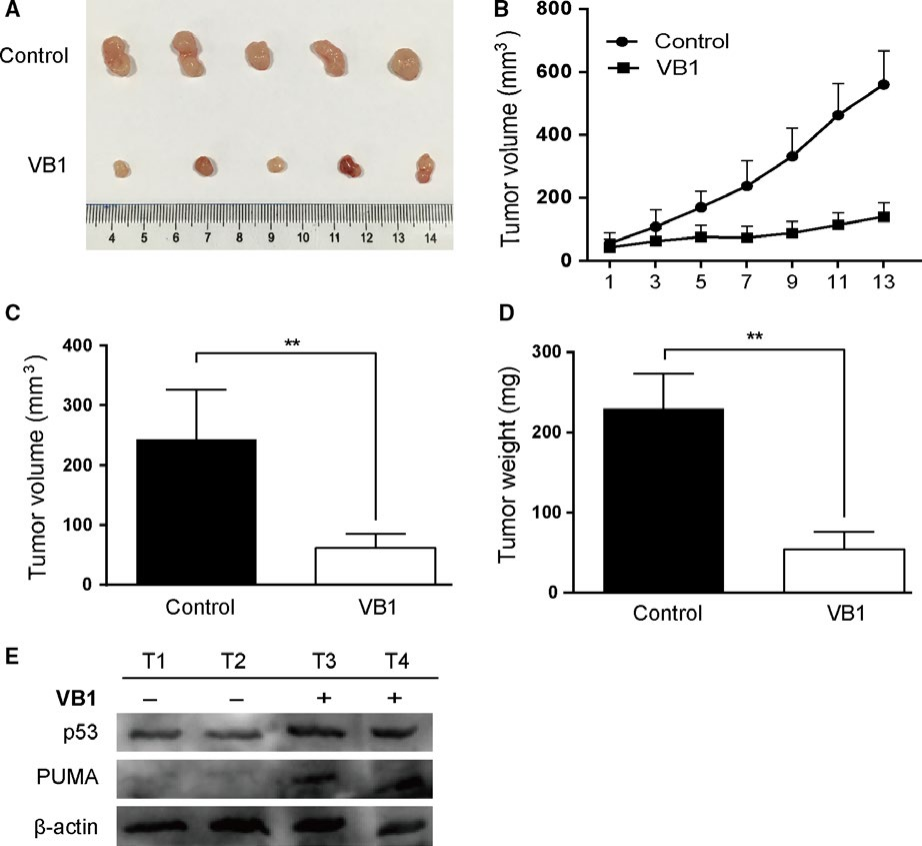
In vivo antitumor activity of VB1. A, Representative tumors at the end of the experiment (on day 15); B, Tumor volumes of control mice and the mice treated with 40 mg/kg VB1 at indicated times (n = 5); C, Calculated tumor volumes at the end of the experiment (on day 15); D, Actual tumor weights at the end of the experiment (on day 15). Nude mice were s.c. injected with 1 × 10^6^ HCT‐116 cells. Once the tumor was measurable, mice were treated with VB1 at 40 mg/kg by i.p. injection every other day for 2 wk. Five mice were used for each group. E, Mice bearing HCT‐116 tumor xenografts were treated with 40 mg/kg of VB1 or normal saline (vehicle control) by i.p. every other day for 2 wk. The tumors (n = 4) were harvested and proteins were extracted for Western blotting analysis. **P <* *0.01, control vs VB1 group

Since VB1 could inhibit cell growth and promote PUMA‐mediated apoptosis in vitro, we further evaluated its in vivo inhibitory effect on tumor growth *in vivo* of HCT‐116 bearing nude mice. As shown in Figure 6A, VB1 significantly suppressed tumor growth compared to the control group after i.p. injection of 40 mg/kg VB1 every other day for 2 weeks. The tumor sizes in VB1‐treated mice were roughly 1/5 of that in vehicle‐treated mice on day 13 (Figure 6B). The weights and volumes of tumors in VB1‐treated group were 75% less than that of tumors in the control group (Figure 6C,D). PUMA and p53 were significantly induced following VB1 treatment in HCT‐116 tumors (Figure 6E). These data show significantly antitumor activity of VB1 in vivo against HCT‐116 tumor xenografts and are consistent with the results achieved in vitro.

## 4 DISCUSSION

Lignans are a group of complex polyphenolic antioxidants in plants and have various pharmacological effects including antioxidant, antimicrobial, and anti‐inflammatory.^46^ An accumulation of evidence has demonstrated that lignans arise as one of the most promising dietary agents for cancer prevention and therapy.^40^, ^47^, ^48^ Previous studies have demonstrated that VB1 acts as a novel antitumor agent for tumor chemoprevention and chemotherapy by targeting cell cycle arrest, antiangiogenesis and apoptosis induction in various cancers both in vitro and in vivo.^41^, ^43^, ^49^, ^50^ Although it has been reported that VB1 induces apoptosis through inhibition of multiple protein kinases and signal transduction pathways,^42^, ^43^ the underlying mechanisms of how VB1 induced apoptosis in colon cancer cells are still not fully clarified. Here, we report the anticancer efficacy of VB1 and associated mechanism in colorectal cancer cells. We demonstrated the anticancer efficacy of VB1 against HCT‐116 and LoVo cells with greater anticancer effect than that of 5‐FU (*P *<* *0.05), a commonly used chemotherapeutic agent in clinic. We are the first to show a role of the p53/PUMA/Bax pathway in VB1‐induced apoptosis in colon cancer WT HCT‐116 cells. The expression of p53‐dependent PUMA directly affects the activation of Bax and the mitochondrial apoptotic pathway.

PUMA plays an important role in mediating apoptosis. In this study, we found that VB1 significantly induced apoptosis and suppressed cell growth in WT HCT‐116 and LoVo cells but not in PUMA‐KO HCT‐116 cells, indicating VB1 induces apoptosis via PUMA‐dependent pathway. Further studies prompted that PUMA promoted VB1 induced Bax activation and the effect of VB1 on apoptosis was significantly attenuated in Bax‐KO HCT‐116 cells. Bim induction was also slightly increased by VB1 in HCT‐116 cells by Western blotting analysis. However, the expressions of Mcl‐1, Bcl‐xl, and Bcl‐2 remained unchanged in HCT‐116 cells. As Bax is almost undetectable in LoVo cells, we speculated that whether Bak (another Bcl‐2 family member like Bax) will play a critical role in mitochondrial outer membrane permeabilization (MOMP) following PUMA induction. As a result, we found that VB1 treatment led to dramatic Bak oligomerization, which is consistent with our hypothesis that after upregulated by p53, PUMA may activate Bak and cause its conformational change in LoVo cells, subsequently, Bak forms oligomer channels in the mitochondrial membrane for cytochrome c release, caspase activation and apoptosis. Further study may be needed to clarify whether other Bcl‐2 family proteins and the inhibitor of apoptosis family of proteins (IAP) such as survivin are involved in cell apoptosis induced by VB1.

Previous studies have shown that NF‐κB pathway 22 and p73 were involved in PUMA activation.^15^, ^16^ However, the phosphorylation level of p65 and the expression of p73 remained unchanged after VB1 treatment. Furthermore, it is well established that FoxO3a can bind to p53‐binding sites in the PUMA promoter,^17^, ^18^, ^19^ our data indicated that FoxO3a was not activated by VB1 in HCT‐116 cells. In addition, the expressions of PUMA and cleaved caspase 3 as well as apoptosis induction by VB1 were significantly reduced in p53‐KO HCT‐116 cells compared to that of WT HCT‐116 cells, suggesting that PUMA induction is through p53‐dependent pathway. However, we also found that VB1 could inhibit cell growth and induce cell apoptosis in mutate p53 colon cancer cells (data not shown). Therefore, other cell‐killing mechanisms of VB1 need further investigation.

Acquired drug resistance is the main reason for poor clinical efficacy of chemotherapy drugs, and the combination of different anticancer mechanisms can effectively reduce the occurrence of drug resistance. Thus, it is urgent to identify and develop effective combination regimens in cancer therapy to prevent and overcome drug resistance. Here, we used the combination of VB1 with chemotherapeutic drugs 5‐FU or NVP‐BZE235. Consistent with our previous studies, 5‐FU induced apoptosis via a p53‐dependent pathway, whereas NVP‐BZE235 induced apoptosis via a p53‐independent pathway.^45^ Our results showed that VB1 increased the expression of p53‐dependent PUMA in HCT‐116 cells. The result may explain the synergistic effect of 5‐FU and VB1 on apoptotic induction due to upregulation of PUMA through p53‐dependent pathway. More importantly, VB1 could further suppress Akt activation when combined with 5‐FU to completely inhibit Akt activation induced by 5‐FU (Figures 5 and S4). NVP‐BZE235 has been reported to upregulate PUMA in HCT‐116 cells in a p53‐independent pathway.^45^ The combination of VB1 and NVP‐BZE235 greatly induced the expression of PUMA via both p53‐dependent and independent pathways. However, whether Akt inhibition contributes to PUMA induction remains unclear and needs to be further investigated.

Taken together, our studies demonstrated the anticancer effects of VB1 against HCT‐116 tumor xenografts in vivo and investigated its mechanisms in colon cancer HCT‐116 and LoVo cells. We also confirmed the indispensable role of p53/PUMA/Bax axis in apoptotic induction and therapeutic efficacy of VB1. Loss of p53, PUMA, or Bax may lead to cancer cells resistance to VB1 in cancer therapy. Therefore, we provide a novel insight for the anticancer mechanism of VB1 and scientific rationale for further development of VB1 as a novel anticancer drug used alone or plus on other chemotherapeutic drugs in the therapy of colorectal cancer clinically.

## CONFLICT OF INTEREST

No conflict of interest.

## References

[1] Tarraga Lopez PJ , Albero JS , Rodriguez‐Montes JA . Primary and secondary prevention of colorectal cancer. Clin Med Insights Gastroenterol. 2014;7:33‐46. 25093007 10.4137/CGast.S14039 PMC4116379

[2] Siegel RL , Miller KD , Jemal A . Cancer statistics, 2016. CA Cancer J Clin. 2016;66:7‐30. 26742998 10.3322/caac.21332

[3] Sorbye H , Cvancarova M , Qvortrup C , Pfeiffer P , Glimelius B . Age‐dependent improvement in median and long‐term survival in unselected population‐based Nordic registries of patients with synchronous metastatic colorectal cancer. Ann Oncol. 2013;24:2354‐2360. 23704193 10.1093/annonc/mdt197

[4] Douillard JY , Cunningham D , Roth AD , et al. Irinotecan combined with fluorouracil compared with fluorouracil alone as first‐line treatment for metastatic colorectal cancer: a multicentre randomised trial. Lancet. 2000;355:1041‐1047. 10744089 10.1016/s0140-6736(00)02034-1

[5] Chian S , Li YY , Wang XJ , Tang XW . Luteolin sensitizes two oxaliplatin‐resistant colorectal cancer cell lines to chemotherapeutic drugs via inhibition of the Nrf2 pathway. Asian Pac J Cancer Prev. 2014;15:2911‐2916. 24761924 10.7314/apjcp.2014.15.6.2911

[6] Lee HC , Ling QD , Yu WC , et al. Drug‐resistant colon cancer cells produce high carcinoembryonic antigen and might not be cancer‐initiating cells. Drug Des Devel Ther. 2013;7:491‐502. 10.2147/DDDT.S45890 PMC3693723 23818760

[7] Wei W , Liu Z , Chen X , Bi F . Chemosensitivity of resistant colon cancer cell lines to lobaplatin, heptaplatin, and dicycloplatin. Int J Clin Pharmacol Ther. 2014;52:702‐707. 24986092 10.5414/CP202023

[8] Zhou J , Li P , Xue X , et al. Salinomycin induces apoptosis in cisplatin‐resistant colorectal cancer cells by accumulation of reactive oxygen species. Toxicol Lett. 2013;222:139‐145. 23916687 10.1016/j.toxlet.2013.07.022

[9] Touil Y , Igoudjil W , Corvaisier M , et al. Colon cancer cells escape 5FU chemotherapy‐induced cell death by entering stemness and quiescence associated with the c‐Yes/YAP axis. Clin Cancer Res. 2013;20:837‐846. 24323901 10.1158/1078-0432.CCR-13-1854 PMC4387277

[10] Chen J , Wang W , Zhang Y , Chen Y , Hu T . Predicting distant metastasis and chemoresistance using plasma miRNAs. Med Oncol. 2014;31:799. 24310813 10.1007/s12032-013-0799-x

[11] Hikisz P , Kilianska ZM . PUMA, a critical mediator of cell death–one decade on from its discovery. Cell Mol Biol Lett. 2012;17:646‐669. 23001513 10.2478/s11658-012-0032-5 PMC6275950

[12] Yu J , Zhang L . No PUMA, no death: implications for p53‐dependent apoptosis. Cancer Cell. 2003;4:248‐249. 14585351 10.1016/s1535-6108(03)00249-6

[13] Yu J , Yue W , Wu B , Zhang L . PUMA sensitizes lung cancer cells to chemotherapeutic agents and irradiation. Clin Cancer Res. 2006;12:2928‐2936. 16675590 10.1158/1078-0432.CCR-05-2429

[14] Zheng X , He K , Zhang L , Yu J . Crizotinib induces PUMA‐dependent apoptosis in colon cancer cells. Mol Cancer Ther. 2013;12:777‐786. 23427294 10.1158/1535-7163.MCT-12-1146 PMC3651803

[15] Ray RM , Bhattacharya S , Johnson LR . Mdm2 inhibition induces apoptosis in p53 deficient human colon cancer cells by activating p73‐ and E2F1‐mediated expression of PUMA and Siva‐1. Apoptosis. 2011;16:35‐44. 20812030 10.1007/s10495-010-0538-0

[16] Ming L , Sakaida T , Yue W , Jha A , Zhang L , Yu J . Sp1 and p73 activate PUMA following serum starvation. Carcinogenesis. 2008;29:1878‐1884. 18579560 10.1093/carcin/bgn150 PMC2722853

[17] Zhang YX , Liu XM , Wang J , et al. Inhibition of AKT/FoxO3a signaling induced PUMA expression in response to p53‐independent cytotoxic effects of H1: a derivative of tetrandrine. Cancer Biol Ther. 2015;16:965‐975. 25893985 10.1080/15384047.2015.1040950 PMC4622009

[18] Dudgeon C , Wang P , Sun X , et al. PUMA induction by FoxO3a mediates the anticancer activities of the broad‐range kinase inhibitor UCN‐01. Mol Cancer Ther. 2010;9:2893‐2902. 20978166 10.1158/1535-7163.MCT-10-0635 PMC2978764

[19] Dey P , Strom A , Gustafsson JA . Estrogen receptor beta upregulates FOXO3a and causes induction of apoptosis through PUMA in prostate cancer. Oncogene. 2014;33:4213‐4225. 24077289 10.1038/onc.2013.384

[20] Sun J , Sun Q , Brown MF , et al. The multi‐targeted kinase inhibitor sunitinib induces apoptosis in colon cancer cells via PUMA. PLoS ONE. 2012;7:e43158. 22912816 10.1371/journal.pone.0043158 PMC3422222

[21] Dudgeon C , Peng R , Wang P , Sebastiani A , Yu J , Zhang L . Inhibiting oncogenic signaling by sorafenib activates PUMA via GSK3beta and NF‐kappaB to suppress tumor cell growth. Oncogene. 2012;31:4848‐4858. 22286758 10.1038/onc.2011.644 PMC3342476

[22] Sun J , Knickelbein K , He K , et al. Aurora kinase inhibition induces PUMA via NF‐kappaB to kill colon cancer cells. Mol Cancer Ther. 2014;13:1298‐1308. 24563542 10.1158/1535-7163.MCT-13-0846 PMC4013266

[23] Siddiqui WA , Ahad A , Ahsan H . The mystery of BCL2 family: Bcl‐2 proteins and apoptosis: an update. Arch Toxicol. 2015;89:289‐317. 25618543 10.1007/s00204-014-1448-7

[24] Deng J . How to unleash mitochondrial apoptotic blockades to kill cancers? Acta Pharm Sin B. 2017;7:18‐26. 28119805 10.1016/j.apsb.2016.08.005 PMC5237704

[25] Gross A . BCL‐2 family proteins as regulators of mitochondria metabolism. Biochim Biophys Acta. 2016;1857:1243‐1246. 26827940 10.1016/j.bbabio.2016.01.017

[26] Ming L , Wang P , Bank A , Yu J , Zhang L . PUMA Dissociates Bax and Bcl‐X(L) to induce apoptosis in colon cancer cells. J Biol Chem. 2006;281:16034‐16042. 16608847 10.1074/jbc.M513587200

[27] Thomas S , Quinn BA , Das SK , et al. Targeting the Bcl‐2 family for cancer therapy. Expert Opin Ther Targets. 2013;17:61‐75. 23173842 10.1517/14728222.2013.733001 PMC3955095

[28] Papaianni E , El Maadidi S , Schejtman A , et al. Phylogenetically distant viruses use the same BH3‐only protein puma to trigger Bax/Bak‐dependent apoptosis of infected mouse and human cells. PLoS ONE. 2015;10:e0126645. 26030884 10.1371/journal.pone.0126645 PMC4452691

[29] Gallenne T , Gautier F , Oliver L , et al. Bax activation by the BH3‐only protein Puma promotes cell dependence on antiapoptotic Bcl‐2 family members. J Cell Biol. 2009;185:279‐290. 19380879 10.1083/jcb.200809153 PMC2700382

[30] Zhang Y , Xing D , Liu L . PUMA promotes Bax translocation by both directly interacting with Bax and by competitive binding to Bcl‐X L during UV‐induced apoptosis. Mol Biol Cell. 2009;20:3077‐3087. 19439449 10.1091/mbc.E08-11-1109 PMC2704159

[31] Chen D , Wei L , Yu J , Zhang L . Regorafenib inhibits colorectal tumor growth through PUMA‐mediated apoptosis. Clin Cancer Res. 2014;20:3472‐3484. 24763611 10.1158/1078-0432.CCR-13-2944 PMC4079733

[32] Yu J , Zhang L , Hwang PM , Kinzler KW , Vogelstein B . PUMA induces the rapid apoptosis of colorectal cancer cells. Mol Cell. 2001;7:673‐682. 11463391 10.1016/s1097-2765(01)00213-1

[33] Jeffers JR , Parganas E , Lee Y , et al. Puma is an essential mediator of p53‐dependent and ‐independent apoptotic pathways. Cancer Cell. 2003;4:321‐328. 14585359 10.1016/s1535-6108(03)00244-7

[34] He K , Zheng X , Zhang L , Yu J . Hsp90 inhibitors promote p53‐dependent apoptosis through PUMA and Bax. Mol Cancer Ther. 2013;12:2559‐2568. 23966620 10.1158/1535-7163.MCT-13-0284 PMC3823684

[35] Brockmann A , Bluwstein A , Kogel A , et al. Thiazolides promote apoptosis in colorectal tumor cells via MAP kinase‐induced Bim and Puma activation. Cell Death Dis. 2015;6:e1778. 26043078 10.1038/cddis.2015.137 PMC4669824

[36] Villunger A , Michalak EM , Coultas L , et al. p53‐ and drug‐induced apoptotic responses mediated by BH3‐only proteins puma and noxa. Science. 2003;302:1036‐1038. 14500851 10.1126/science.1090072

[37] Han JW , Flemington C , Houghton AB , et al. Expression of bbc3, a pro‐apoptotic BH3‐only gene, is regulated by diverse cell death and survival signals. Proc Natl Acad Sci USA. 2001;98:11318‐11323. 11572983 10.1073/pnas.201208798 PMC58727

[38] Liu CJ , Zhang XL , Luo DY , et al. Exogenous p53 upregulated modulator of apoptosis (PUMA) decreases growth of lung cancer A549 cells. Asian Pac J Cancer Prev. 2015;16:741‐746. 25684518 10.7314/apjcp.2015.16.2.741

[39] Adlercreutz H . Phyto‐oestrogens and cancer. Lancet Oncol. 2002;3:364‐373. 12107024 10.1016/s1470-2045(02)00777-5

[40] Thompson LU , Chen JM , Li T , Strasser‐Weippl K , Goss PE . Dietary flaxseed alters tumor biological markers in postmenopausal breast cancer. Clin Cancer Res. 2005;11:3828‐3835. 15897583 10.1158/1078-0432.CCR-04-2326

[41] Zhou Y , Liu YE , Cao J , et al. Vitexins, nature‐derived lignan compounds, induce apoptosis and suppress tumor growth. Clin Cancer Res. 2009;15:5161‐5169. 19671865 10.1158/1078-0432.CCR-09-0661 PMC2752044

[42] Wang J , Zheng X , Zeng G , Zhou Y , Yuan H . Purified vitexin compound 1 inhibits growth and angiogenesis through activation of FOXO3a by inactivation of Akt in hepatocellular carcinoma. Int J Mol Med. 2014;33:441‐448. 24337611 10.3892/ijmm.2013.1587

[43] Liu LH , Zhou YJ , Ding L , Zhang SZ , Sun J , Cao JG . Induction of apoptosis by VB1 in breast cancer cells: the role of reactive oxygen species and Bcl‐2 family proteins. Int J Mol Med. 2014;33:423‐430. 24276280 10.3892/ijmm.2013.1567

[44] Xin H , Kong Y , Wang Y , et al. Lignans extracted from Vitex negundo possess cytotoxic activity by G2/M phase cell cycle arrest and apoptosis induction. Phytomedicine. 2013;20:640‐647. 23562365 10.1016/j.phymed.2013.02.002

[45] Wang H , Zhang L , Yang X , et al. PUMA mediates the combinational therapy of 5‐FU and NVP‐BEZ235 in colon cancer. Oncotarget. 2015;6:14385‐14398. 25965911 10.18632/oncotarget.3775 PMC4546474

[46] Adlercreutz H . Lignans and human health. Crit Rev Clin Lab Sci. 2007;44:483‐525. 17943494 10.1080/10408360701612942

[47] Tang SH , He RR , Huang T , et al. The protective effect of Schisandra lignans on stress‐evoked hepatic metastases of P815 tumor cells in restraint mice. J Ethnopharmacol. 2011;134:141‐146. 21130851 10.1016/j.jep.2010.11.070

[48] Bergman Jungestrom M , Thompson LU , Dabrosin C . Flaxseed and its lignans inhibit estradiol‐induced growth, angiogenesis, and secretion of vascular endothelial growth factor in human breast cancer xenografts in vivo. Clin Cancer Res. 2007;13:1061‐1067. 17289903 10.1158/1078-0432.CCR-06-1651

[49] Wang JG , Zheng XX , Zeng GY , Zhou YJ , Yuan H . Purified vitexin compound 1 induces apoptosis through activation of FOXO3a in hepatocellular carcinoma. Oncol Rep. 2014;31:488‐496. 24247909 10.3892/or.2013.2855

[50] Deng J , Zhang Y , Tan Z . Proliferation and apoptosis of choriocarcinoma cell JEG‐3 induced by VB2 and its in vitro mechanism. Zhong Nan Da Xue Xue Bao Yi Xue Ban. 2013;38:476‐482. 23719531 10.3969/j.issn.1672-7347.2013.05.006

