# Supplementary figures and images for: Purified vitexin compound 1, a new neolignan isolated compound, promotes PUMA‐dependent apoptosis in colorectal cancer
Source: Cancer Med. 2018 Nov 6;7(12):6158–69. doi: 10.1002/cam4.1769 (PMC6308053; doi:10.1002/cam4.1769)

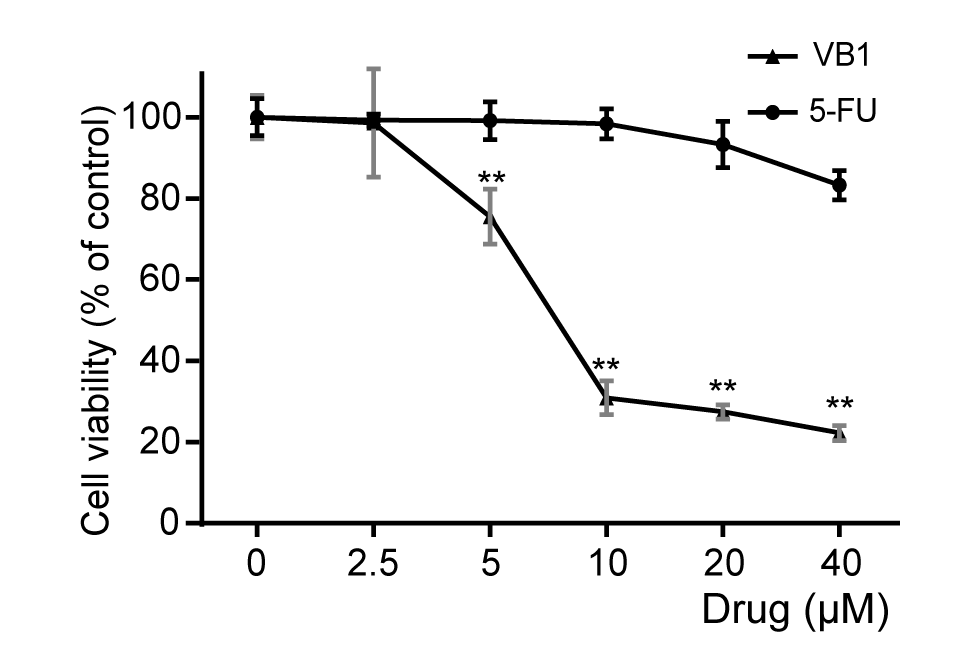

Supplement: Supplementary file 1 [file CAM4-7-6158-s001.tif]

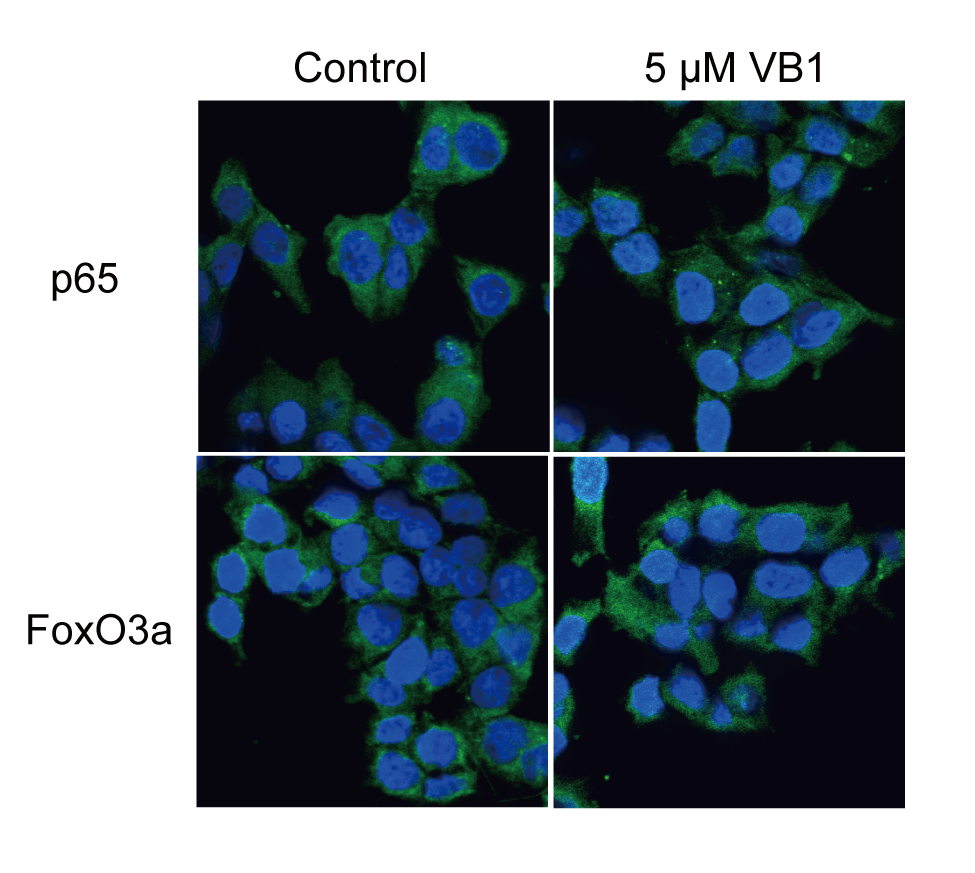

Supplement: Supplementary file 2 [file CAM4-7-6158-s002.tif]

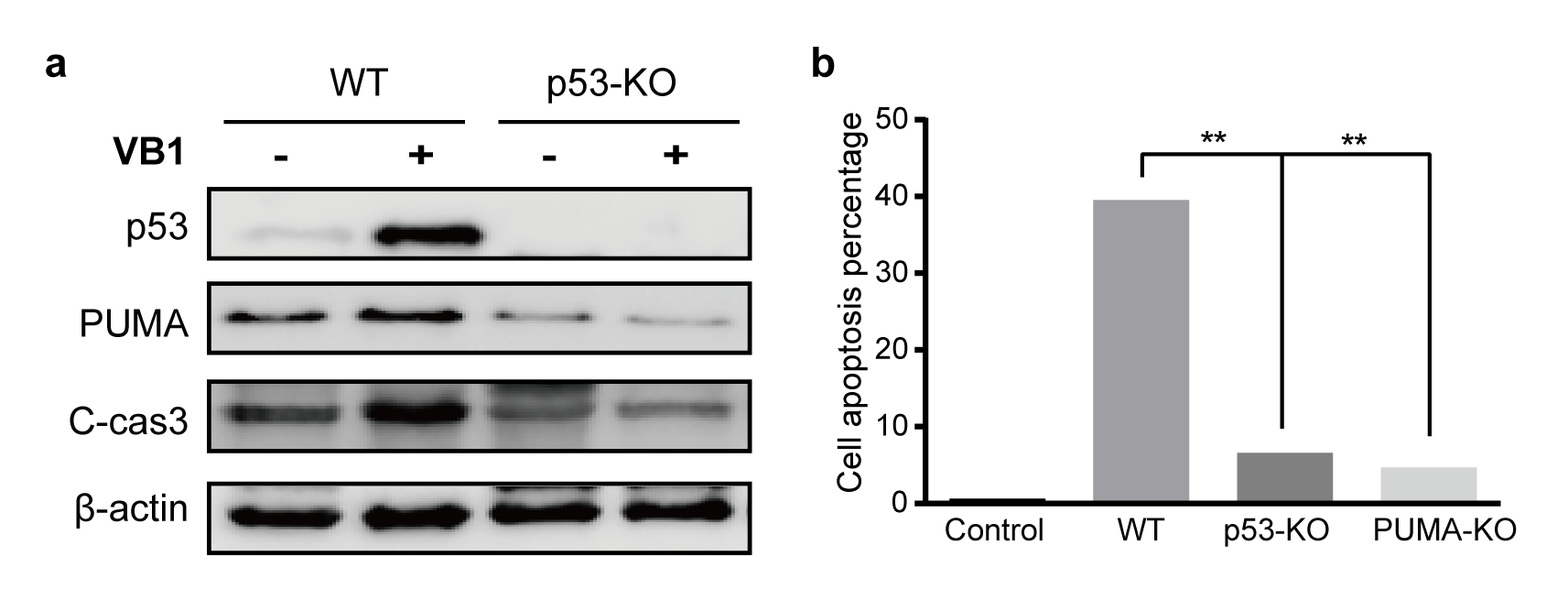

Supplement: Supplementary file 3 [file CAM4-7-6158-s003.tif]

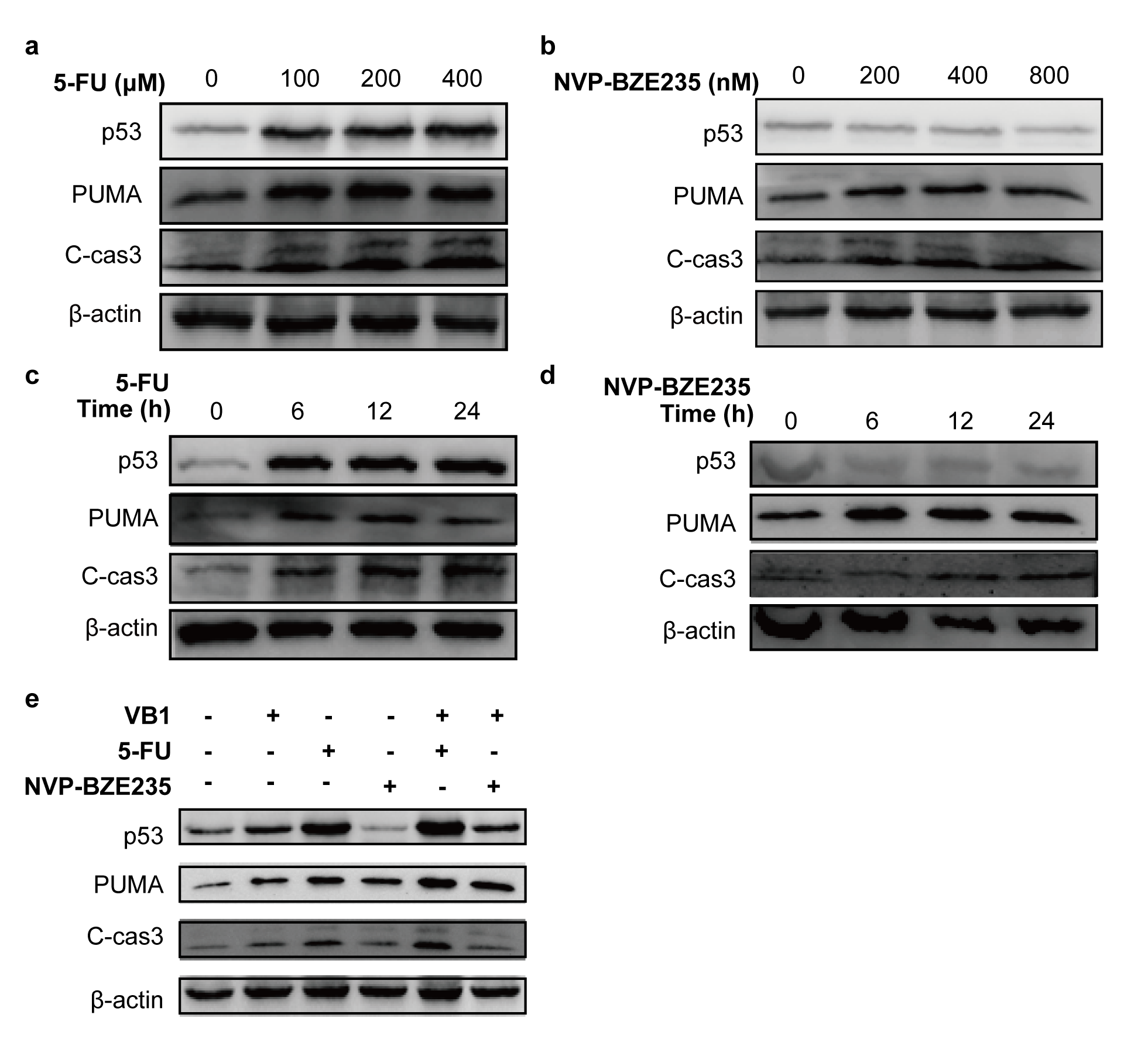

Supplement: Supplementary file 4 [file CAM4-7-6158-s004.tif]

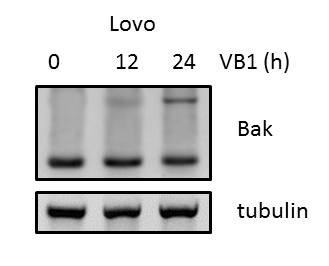

Supplement: Supplementary file 5 [file CAM4-7-6158-s005.tif]

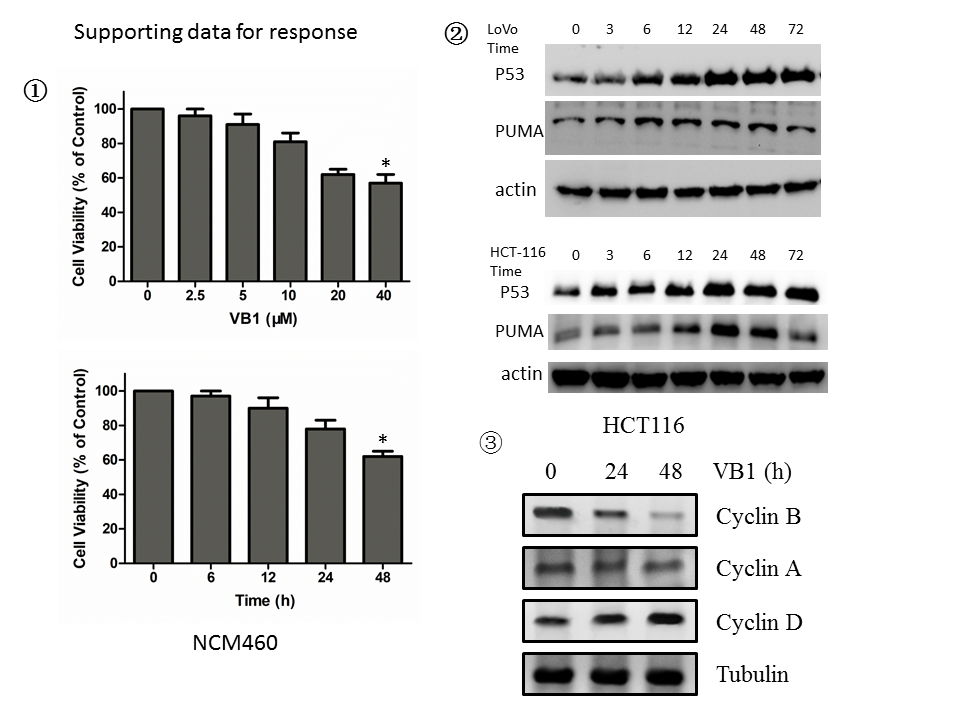

Supplement: Supplementary file 7 [file CAM4-7-6158-s007.tif]
