# Supplementary material for: Purified vitexin compound 1, a new neolignan isolated compound, promotes PUMA‐dependent apoptosis in colorectal cancer
Source: Cancer Med. 2018 Nov 6;7(12):6158–69. doi: 10.1002/cam4.1769 (PMC6308053; doi:10.1002/cam4.1769)
Supplement: Supplementary file 6 [file CAM4-7-6158-s006.docx]

**Supporting information : Figure Legends**

**Fig. S1** Comparison of the effects of VB1 and 5-FU on cell viability in HCT-116 cells by CCK-8 analysis. The cells were treated with VB1 or 5-FU at 0, 2.5, 5, 10, 20, or 40 µM for 72 h. Data represent the mean ± SD of four independent experiments. ***P*< 0.01, VB1 vs 5-FU.

**Fig. S2** Effects of VB1 on the translocation of p65 and FoxO3a in HCT-116 cells by immunofluorescence analysis. The cells were treated with 10 µM VB1 for 12 h and nuclei was stained by 4',6-diamidino-2-phenylindole (DAPI).

**Fig. S3** Effect of VB1 on the expressions of p53, PUMA and cleaved caspase 3 and apoptotic induction in HCT-116 cells. **a** Western blotting analysis: WT, p53-KO HCT-116, PUMA-KO HCT-116 cells were treated with 10 µM VB1 for 24 h. **b** The percentage of apoptotic cells were calculated from FACS analysis in Fig. 3g. Data represent the mean ± SD of three independent experiments. ***P*< 0.01, WT vs p53-KO or PUMA-KO.

**Fig. S4.** Effects of VB1 and 5-FU or NVP-BZE235 alone or in combination on the expressions of p53, PUMA, and C-cas3. **a** The cells were treated with 5-FU at 0, 100, 200, or 400 µM for 24 h. **b** The cells were treated with NVP-BZE235 at 0, 200, 400, or 800 nM for 24 h. **c** The cells were treated with 200 µM 5-FU for 0, 6, 12, or 24 h. **d** The cells were treated with 400 nM NVP-BZE235 for 0, 6, 12, or 24 h. **e** The cells were treated with 10 µM VB1 ± 200 µM 5-FU or ± 400 nM NVP-BZE235.

**Fig. S5.** Effect of VB1 on the expressions of Bak in LoVo cells. **a** The cells were treated with 10 µM VB1 for 24 or 48 hours and Bak was detected by western blotting. Tubulin was used as a loading control.
